# Intravoxel incoherent motion diffusion-weighted imaging to differentiate hepatocellular carcinoma from intrahepatic cholangiocarcinoma

**DOI:** 10.1038/s41598-020-64804-9

**Published:** 2020-05-07

**Authors:** Juan Peng, Jing Zheng, Cui Yang, Ran Wang, Yi Zhou, Yun-Yun Tao, Xue-Qin Gong, Wei-Cheng Wang, Xiao-Ming Zhang, Lin Yang

**Affiliations:** 1 Department of Radiology, Medical Research Center, Affiliated Hospital of North Sichuan Medical College, Nanchong, Sichuan 637000 P. R. China; 2 Department of Radiology, Sichuan Provincial People’s Hospital Jinniu Hospital,Chengdu Jinniu District People’s Hospital, Chengdu, Sichuan 610007 P. R. China

**Keywords:** Liver cancer, Gastroenterology, Cancer, Cancer

## Abstract

The present study aimed to explore the value of intravoxel incoherent motion diffusion-weighted imaging (IVIM-DWI) in differentiating hepatocellular carcinoma (HCC) from intrahepatic cholangiocarcinoma (ICC). This study included 65 patients with malignant hepatic nodules (55 with HCC, 10 with ICC), and 17 control patients with normal livers. All patients underwent IVIM-DWI scans on a 3.0 T magnetic resonance imaging (MRI) scanner. The standard apparent diffusion coefficient (ADC), pure diffusion coefficient (D_slow_), pseudo-diffusion coefficient (D_fast_), and perfusion fraction (f) were obtained. Differences in the parameters among the groups were analysed using one-way ANOVA, with p < 0.05 indicating statistical significance. Receiver operating characteristic (ROC) curve analysis was used to compare the efficacy of each parameter in differentiating HCC from ICC. ADC, D_slow_, D_fast_, f significantly differed among the three groups. ADC and D_slow_ were significantly lower in the HCC group than in the ICC group, while D_fast_ was significantly higher in the HCC group than in the ICC group; f did not significantly differ between the HCC and ICC groups. When the cut-off values of ADC, D_slow_, and D_fast_ were 1.27 × 10^−3^ mm^2^/s, 0.81 × 10^−3^ mm^2^/s, and 26.04 × 10^−3^ mm^2^/s, respectively, their diagnostic sensitivities for differentiating HCC from ICC were 98.18%, 58.18%, and 94.55%, their diagnostic specificities were 50.00%, 80.00%, and 80.00%, and their areas under the ROC curve (AUCs) were 0.687, 0.721, and 0.896, respectively. D_fast_ displayed the largest AUC value. IVIM-DWI can be used to differentiate HCC from ICC.

## Introduction

Hepatocellular carcinoma (HCC) and intrahepatic cholangiocarcinoma (ICC) are the common primary liver cancers (PLCs) worldwide. Because the therapeutic strategies and prognosis of HCC and ICC are quite different, accurate differentiation is very important^1^. However, distinguishing HCC from ICC by using conventional imaging methods is often fairly difficult because these diseases have similar imaging features^2^. Diffusion-weighted imaging (DWI) is a quantitative method used to detect the metabolic functions of live tissues by measuring the apparent diffusion coefficient (ADC)^3–5^. However, DWI cannot distinguish between the diffusion of water molecules and the perfusion of blood. Specifically, intravoxel incoherent motion diffusion-weighted imaging (IVIM-DWI) can simultaneously quantify the diffusion of water molecules and microcirculation perfusion in living tissues, and thus compensates for the limitations of traditional DWI^6–9^. Among the parameters used by IVIM-DWI, the pure diffusion coefficient (D_slow_) reflects the diffusion of pure water molecules, the pseudo-diffusion coefficient (D_fast_) reflects the diffusion movement of capillary microcirculation perfusion, and the perfusion fraction (f) represents the volume ratio between the perfusion effect of local microcirculation and the overall diffusion effect.

IVIM-DWI technology has been applied to the liver with the primary goals of identifying benign and malignant hepatic nodules^10–18^, determining histologic grades^19–21^, assessing the treatment response of HCC^22–25^, grading cirrhosis^11,26–34^ and evaluating non-alcoholic fatty liver disease^35–37^. However, the application of IVIM-DWI in the differential diagnosis of malignant nodules with different pathological properties in the liver has been rarely explored. The aim of the present study was to explore the value of IVIM-DWI in differentiating HCC from ICC.

## Materials and Methods

### Patients

The present study was approved by the Ethics of affiliated hospital of north Sichuan medical college, written informed consent was obtained for all individual participants, and all methods were performed in accordance with the relevant guidelines and regulations. Sixty-five patients with PLCs were enrolled in this study, including 52 males and 13 females, with ages ranging from 18 to 78 years and an average age of 51.2 ± 14.6 years. All patients underwent surgery, and their tumour statuses were confirmed by pathological evaluation. Normal liver tissues from another 17 patients served as the control group.

### MRI

Using a GE Discovery MR750 3.0 T superconducting MRI scanner and a body-specific 32-channel phased-array coil (GE Medical Systems, Milwaukee, Wis., USA), all patients successively underwent axial breath-hold fat-suppressed T1-weighted imaging (T1WI), respiratory-triggered fat-suppressed T2-weighted imaging (T2WI), IVIM-DWI, and multiphase dynamic contrast-enhanced MRI. Before the scan, the patients fasted for 4 h and went through respiratory training. The scan covered the area from the top of the diaphragm to the lower edge of the liver (a scan of the entire liver). The IVIM-DWI sequence was selected based on nine different b-values (b = 0, 20, 40, 80, 100, 200, 400, 800, and 1,000 s/mm^2^) with a repetition time/echo time (TR/TE) of 3,529 ms/60.8 ms, matrix of 128 × 160, field of view (FOV) of 36 cm × 36 cm − 40 cm × 40 cm and layer thickness/layer spacing of 5 mm/0.5 mm. During the multiphase dynamic contrast-enhanced MRI scan, the contrast agent Gd-DTPA (Bayer AG, Mullerstrasse 178, Berlin-Wedding 13353, Germany) was intravenously injected into the dorsal hand vein of each patient at a speed of 2.5 ml/s and a dose of 0.2 ml/kg. After the injection of the contrast agent, images were collected in the following phases: hepatic arterial phase, portal venous phase, late portal venous phase, and delayed phase (axial and coronal planes).

### Data measurement

The images were transferred to the GE ADW4.6 post-processing workstation (GE Medical Systems, Milwaukee, Wis., USA), and image analysis was performed using Function-MADC software (GE Medical Systems, Milwaukee, Wis., USA). Pseudo-colour maps of the standard ADC, D_slow_, D_fast_, and f were generated. Regions of interest (ROIs) were placed in the largest solid areas of the lesions (avoiding regions of necrosis and haemorrhage in the tumours), and the IVIM-DWI parameters were measured.

### Statistical analysis

All data were subjected to statistical analysis using SPSS 21.0 software. One-way ANOVA was used to analyse the differences in the parameters among different groups. When p < 0.05, the difference was considered statistically significant. The receiver operating characteristic (ROC) curve was used to compare the efficacy of each parameter in differentiating HCC from ICC.

## Results

**Figure 1:**
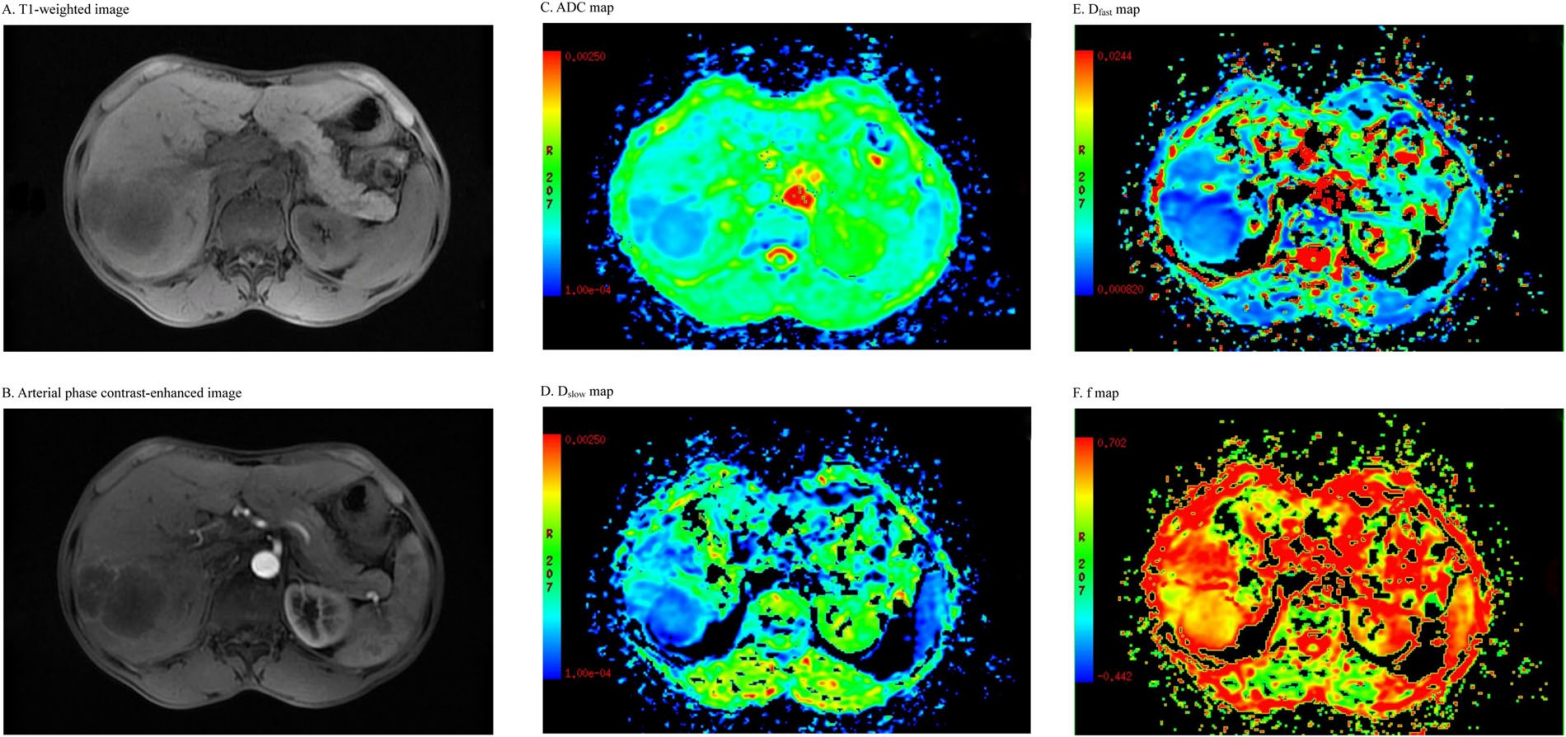
Axial MRI images of an HCC patient. (**A**): A T1-weighted image shows a hypointensive lesion in the right lobe of the liver. (**B**): An arterial phase contrast-enhanced image shows an inhomogeneous enhanced lesion. (**C**): ADC map. (**D**): D_slow_ map. (**E**): D_fast_ map. (**F**): f map.

**Figure 2:**
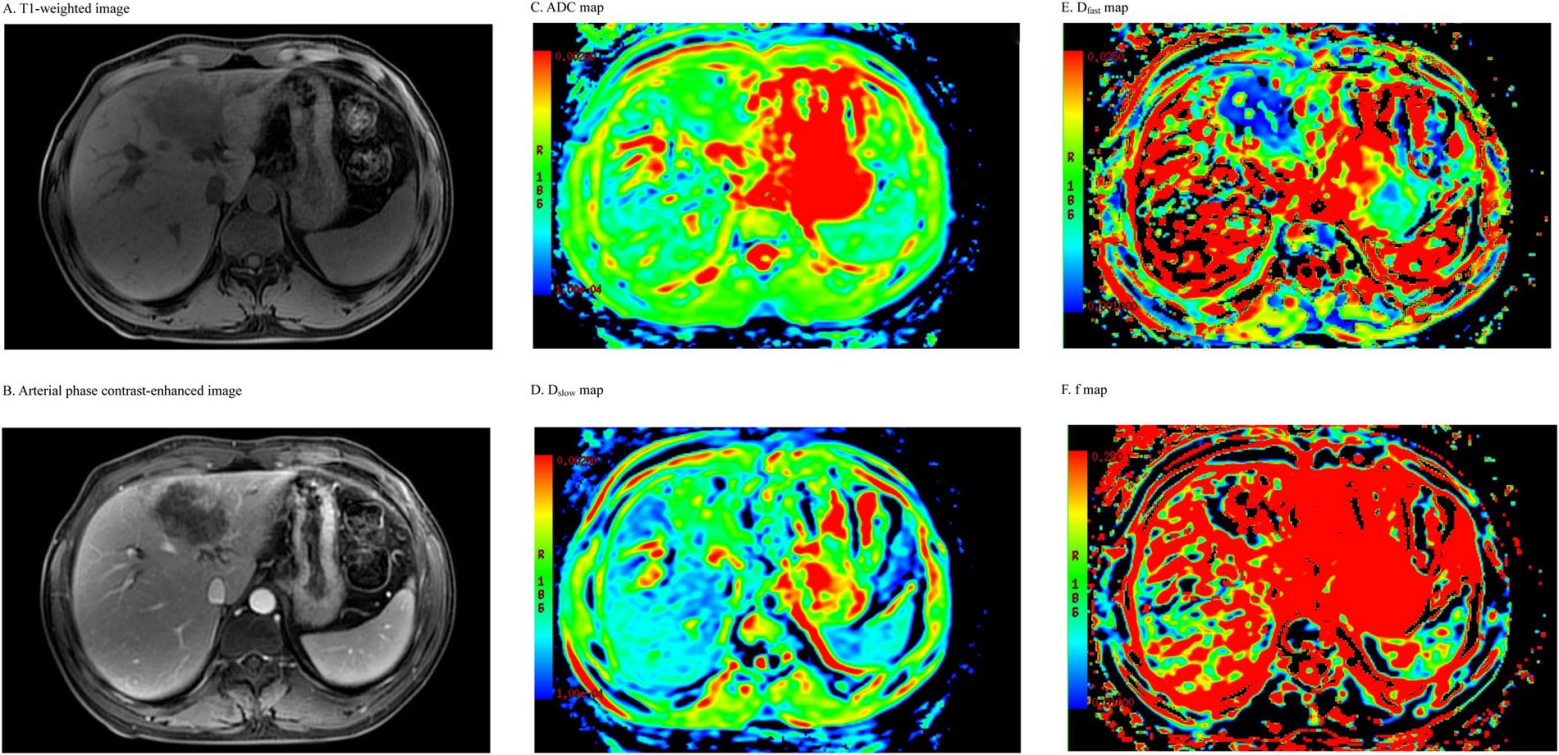
MR images of an ICC patient. (**A**): A T1-weighted image shows a hypointense lesion in the left lobe of the liver. (**B**): An arterial phase contrast-enhanced image shows an inhomogeneous enhanced lesion with biliary obstruction. (**C**): ADC map. (**D**): D_slow_ map. (**E**): D_fast_ map. (**F**): f map.

**Figure 3:**
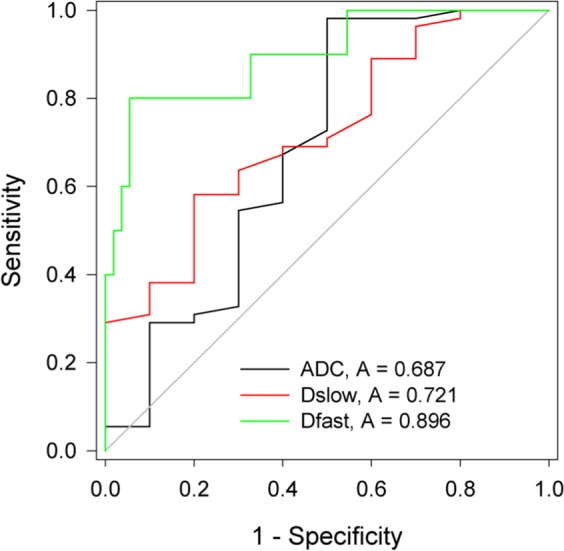
Receiver operating characteristic (ROC) curves of the IVIM-DWI parameters for differentiating HCC and ICC. The area under the curve (AUC) for D_fast_ was higher than those for the ADC and D_slow_.

Among the 65 cases of PLCs, 55 were HCCs, and 10 were ICCs. Forty-eight patients had cirrhosis. The Child-Pugh score of liver function showed that 55 of the cases were grade A while 10 cases were grade B. The ADC, D_slow_, D_fast_ and f all significantly differed among the HCC tissues, ICC tissues, and normal liver tissues (all p < 0.05). The ADC, D_slow_, D_fast_, and f of the HCC tissues were all lower than the ADC, D_slow_, D_fast_ and f of the normal liver tissues (all p < 0.05). D_fast_ and f of the ICC tissues were lower than those of the normal liver tissues, but no significant differences were found in the other parameters between the two groups. The ADC and D_slow_ of HCC tissues were both significantly lower than those of ICC tissues (all p < 0.05), whereas D_fast_ of the HCC tissues was significantly higher than that of the ICC tissues (p < 0.05). Additionally, the f did not significantly differ between the HCC and ICC tissues (p > 0.05). When the cut-off values of the ADC, D_slow_, and D_fast_ were 1.27 × 10^−3^ mm^2^/s, 0.81 × 10^−3^ mm^2^/s, and 26.04 × 10^−3^ mm^2^/s, respectively, their diagnostic sensitivities for differentiating HCC from ICC were 98.18%, 58.18%, and 94.55%, respectively, their diagnostic specificities were 50.00%, 80.00%, and 80.00%, respectively, and their area under the curve (AUC) values were 0.687, 0.721, and 0.896, respectively; Dfast displayed the largest AUC (Tables 1 and 2) (Figs. 1–3). Among the 55 cases of HCC, 18 were World Health Organization (WHO) grade I, 22 were grade II, and 15 were grade III. The ADC and D_slow_ of HCC were significantly negatively correlated with the histological grades of the lesions (all p < 0.05), and the f of HCC was significantly positively correlated with the histological grades of the corresponding lesions (all p < 0.05).

**Table 1:** Comparison of the IVIM-DWI parameters among HCC, ICC, and control groups.

	ADC	D_slow_	D_fast_	f
HCC group	1.01 ± 0.19^a^	0.81 ± 0.18^a^	35.36 ± 6.77^a^	0.18 ± 0.06^a^
ICC group	1.17 ± 0.27^b^	0.98 ± 0.22^b^	24.16 ± 5.87^b^	0.15 ± 0.03^a^
Control group	1.22 ± 0.03^b^	1.10 ± 0.05^b^	65.32 ± 14.93^c^	0.22 ± 0.03^b^
F	10.896	20.327	90.684	6.456
p	0.000	0.000	0.000	0.003

Note: The units for the ADC, D_slow_, and D_fast_ are all 10^−3^ mm^2^/s. Different letters at the upper right corners of the numbers indicate significant difference between the groups.

**Table 2:** The diagnostic performance of the IVIM-DWI parameters for the characterization of malignant liver nodules.

Parameters	Cut-off	Sensitivity (%)	Specificity (%)	AUC	95%CI
ADC	1.27	98.18	50.00	0.687	0.469–0.906
D_slow_	0.81	58.18	80.00	0.721	0.557–0.885
D_fast_	26.04	94.55	80.00	0.896	0.782–1.000

Note: 95%CI: 95% confidence interval.

## Discussion

IVIM-DWI acquires DWI images with multiple b-values and uses a bi-exponential pattern to extract quantitative information that reflects local tissue water molecule diffusion and microcirculation perfusion^6–9^. The application of IVIM-DWI to identify benign and malignant hepatic nodules has been proven to be important by previous researchers^10–18^. However, studies on the differential diagnosis of malignant hepatic nodules with different pathological properties by IVIM-DWI are very limited^10,17,38^. Watanabe H *et al*.^12^ performed IVIM-DWI scans on a total of 120 hepatic nodules in 74 patients. The results showed that the true molecular-diffusion coefficient (D) and ADC of malignant nodules were significantly lower than those of benign nodules. The D and ADC of hepatic cysts were higher than those of hemangiomas, but the D and ADC did not differ between metastasis and HCC. The ADC of benign and malignant lesions had significantly greater AUCs than the D of the two types of lesions. Choi IY *et al*.^17^ performed IVIM-DWI scanning of 161 liver nodule cases. The results showed that the D_slow_ values of HCC were obviously lower than those of ICC, and the f values of HCC were higher than those of ICC and metastasis. No differences were found among the ADC values of malignant nodules with different pathological properties. Among all of the parameters used to identify malignant nodules, D_slow_ displayed the largest AUC, and there were significant positive correlations between the f value and the enhancement fraction and between the f value and the relative enhancement. A study by Wei *et al*.^38^ found that the ADC and D_slow_ can be beneficial for the differential diagnosis of HCC and ICC. D_slow_ showed a better discriminatory ability than the ADC, whereas D_fast_ and f displayed no differentiating power for ICC and HCC.

Data obtained from our study found significant differences among some of the IVIM-DWI parameters, including ADC, D_slow_, and D_fast_ between HCC and ICC. The ADC and D_slow_ of HCC tissues were obviously lower than those of ICC tissues, which is consistent with the results reported by Choi^17^ and Wei *et al*.^38^. The D_fast_ value of HCC tissues was significantly higher than that of ICC tissues, which may be associated with the rich blood supply inherent to HCC, while the blood supply of ICC is poor^39^. This result was not observed by Wei *et al*.^38^. The differential efficacy of D_fast_ for malignant hepatic nodules with different pathological features is higher than those of the ADC and D_slow_, suggesting that D_fast_ can better reflect the changes in the tissue microstructure compared to the ADC and D_slow_. No significant differences in the f values were found between HCC and ICC in this study in contrast to D_fast_, which reflects changes in tissue perfusion. This might be because the D_fast_ value and the f value represent different aspects of perfusion, with D_fast_ representing the flow rate of blood in the microvessels and the f value representing the blood-carrying capacity of the capillaries^40^.

Most studies have reported no differences in the IVIM-DWI parameters between HCC and liver metastases^12,41^. However, Choi *et al*.^17^ reported that the f value of HCC tissue is higher than that of metastatic tissue. The primary results from our study demonstrated that except for the slightly higher D_fast_ values in HCC than those in metastatic cancer, the other parameters, including the ADC, D_slow_, and f, showed no difference between HCC and metastatic cancer. These inconsistencies could be associated with the different primary lesions and the altered cell density and microcirculation in the metastatic lesions.

Woo *et al*.^19^ acquired DWI scans of 42 HCC cases based on eight selected b-values (0–800 s/mm^2^). The results showed that the diffusion coefficient (D) and ADC of high-grade HCC were significantly lower than those of low-grade HCC. Both the D and ADC were significantly associated with the pathological grades of the tumours. Regarding high or low grades of HCC, the AUC of the D value was larger than that of the ADC value, and the percentage of arterial enhancement was associated with the f value. The results of the present study showed that the ADC and D_slow_ of HCC tissues are negatively correlated with the histological grades of the lesions, whereas the f is positively correlated with the histological grades of the lesions, suggesting that IVIM-DWI can be used to evaluate the preoperative histological grades of HCC. The findings of the present study are essentially consistent with the results reported by Woo *et al*.

IVIM-DWI assessment of liver nodules can be influenced by certain pathological conditions such as liver fibrosis. Studies have shown that cirrhosis leads to restricted water molecule diffusion and therefore reduced blood perfusion. Luciani *et al*.^34^ discovered that compared with those of normal liver tissues, the ADC and D_fast_ of cirrhotic tissue were significantly lower, but D_slow_ and f remained similar among the two types of tissue. A report by Patel *et al*.^42^ suggested that the ADC, D_slow_, D_fast_, and f were all lower in the cirrhotic group than those in the non-cirrhotic group. In this study, most patients in the HCC group had cirrhosis; thus, we used normal livers as negative controls. The results revealed that the ADC, D_slow_, D_fast_, and f of HCC tissues were lower than those of normal liver tissues.

In the IVIM-DWI model, multiple b-values are used to perform a double-exponential fit on the DWI signal, which requires at least four different b-values (including b = 0 s/mm^2^) to complete the calculation of related parameters. In the application of IVIM-DWI, a considerable discrepancy was found in the selection of b-values by different researchers^43–49^. At present, the number of b-values used in most studies is approximately eight to 14^15,34,42,48^. In the IVIM-DWI model, when the selected b-value is high, the attenuation of the signal basically reflects the pure water molecule diffusion, but when a low b-value is selected, the attenuation of the signal is more sensitive to the perfusion effect of the local microcirculation capillaries. The higher the number of b-values is, the longer the image acquisition time required. Therefore, optimizing the number and distribution of b-values and reducing the errors of IVIM-DWI parameter measurement are very important. Dyvorne *et al*.^48^ investigated the impact of the number of b-values on IVIM-DWI parameters through the comparison and analysis of 16 b-values and measurement results of four optimized b-values of a reference group. Their results indicated that the accuracy and repeatability of the IVIM-DWI parameters were not significantly affected, and the latter approach actually reduced the image acquisition time considerably. The b-values in our study were selected according to the considerations set forth in previous literature^17,45,49^, and small adjustments were made in accordance with the practical situation of our unit. A total of nine b-values were selected, of which five were lower values. The time of collection was approximately 6 min and 30 s. In future research, the quantity and distribution of b-values should be selected based on the different tissues examined and optimized through in-depth research^43^.

The present study has several limitations. First, a limited number of ICC cases compared to the number of HCC cases were included in the sample groups. As a next step, we will continue the study with an increased number of ICC cases. Second, this study did not examine difference between the IVIM-DWI parameters of benign liver lesions and those of malignant lesions or investigate differences in IVIM-DWI parameters between HCC and other liver malignancies, which will be studied in a related project in the future after more cases are collected. Third, the relationship between IVIM-DWI parameters and the prognosis of the patients was not analysed. We plan to follow-up the prognosis of the patients and carry out relevant analyses in subsequent work.

In conclusion, IVIM-DWI can provide quantitative information reflecting water molecule diffusion and microcirculation perfusion in the local tissue. Furthermore, IVIM-DWI can be used for the differential identification of malignant nodules with different pathological properties in the liver.
